# Vegetable-Based Diets for Chronic Kidney Disease? It Is Time to Reconsider

**DOI:** 10.3390/nu11061263

**Published:** 2019-06-04

**Authors:** Aleix Cases, Secundino Cigarrán-Guldrís, Sebastián Mas, Emilio Gonzalez-Parra

**Affiliations:** 1 Medicine Department, Universitat de Barcelona, Institut d’Investigacions Biomèqiques August Pi i Sunyer, 08036 Barcelona, Spain; acases@clinic.cat; 2 Servicio de Nefrología, Hospital da Costa, 27880 Burela, Spain; Secundino.Cigarran.Guldris@sergas.es; 3 Servicio de Nefrología, Fundación Jiménez Díaz, 28040 Madrid, Spain; smas@fjd.es; 4 Centro de investigación en Red de Diabetes y Enfermedades Metabólicas Asociadas (CIBERDEM), 28029 Madrid, Spain; 5 Red de Investigación Renal (RedinRen), 28029 Madrid, Spain

**Keywords:** CKD, vegetable-based diet, hyperkalemia, fiber, gut microbiota, dietary acid load, uremic toxins, phosphorus

## Abstract

Traditional dietary recommendations to renal patients limited the intake of fruits and vegetables because of their high potassium content. However, this paradigm is rapidly changing due to the multiple benefits derived from a fundamentally vegetarian diet such as, improvement in gut dysbiosis, reducing the number of pathobionts and protein-fermenting species leading to a decreased production of the most harmful uremic toxins, while the high fiber content of these diets enhances intestinal motility and short-chain fatty acid production. Metabolic acidosis in chronic kidney disease (CKD) is aggravated by the high consumption of meat and refined cereals, increasing the dietary acid load, while the intake of fruit and vegetables is able to neutralize the acidosis and its deleterious consequences. Phosphorus absorption and bioavailability is also lower in a vegetarian diet, reducing hyperphosphatemia, a known cause of cardiovascular mortality in CKD. The richness of multiple plants in magnesium and vitamin K avoids their deficiency, which is common in these patients. These beneficial effects, together with the reduction of inflammation and oxidative stress observed with these diets, may explain the reduction in renal patients’ complications and mortality, and may slow CKD progression. Finally, although hyperkalemia is the main concern of these diets, the use of adequate cooking techniques can minimize the amount absorbed.

## 1. Introduction

Nephrologists classically do not recommend vegetable-based diets since they have been considered nutritionally inadequate and dangerous for the management of patients with chronic kidney disease (CKD), due to their high potassium (K) content. But vegetable-based diets are sufficient for a balanced protein intake, and for several reasons have shown to reduce mortality in non-CKD patients [1,2]. Although it is a common belief that plant-based diets are deficient in all the essential amino acids, it has been shown that it is not necessarily so [3]. In fact, the European Prospective Investigation into Cancer and Nutrition (EPIC)-Oxford and California Seventh-day Adventists cohorts support the idea that well-balanced and diverse vegetable-based diets can be nutritionally adequate [4] and beneficial [1,2].

**Figure 1:**
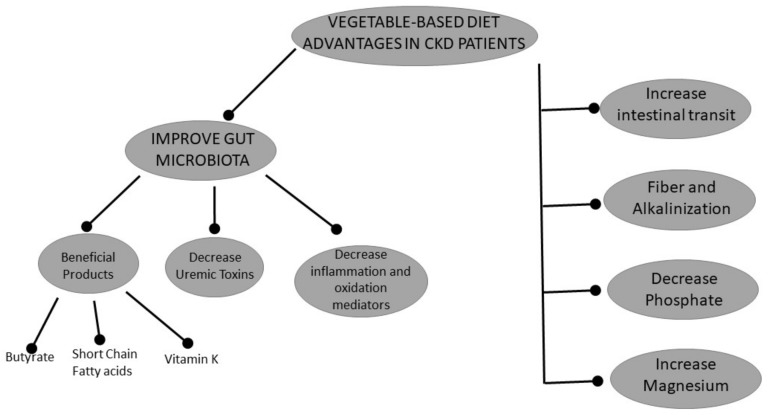
Scheme of the beneficial effects of a plant-based diet, through its direct nutritional contribution or the changes it produces in the intestinal microbiota.

Plant-based diets have been prescribed in CKD without any adverse effects. Thus, it is unlikely that malnutrition or protein-energy wasting will occur with these diets in renal patients. A study in CKD stage 3–4 patients in which a vegan diet, composed of a prespecified combination of cereals and legumes, to ensure the intake of all essential amino acids, demonstrated no signs of nutritional deficiency after an average follow-up of 13 months; the authors proposed this diet as a cheaper and more palatable alternative to conventional low-protein diets in this population [5]. CKD patients following plant-based diets do not need supplementation with keto-analogues or essential amino acids if they consume at least 0.6 g/kg/day of protein [6], while unrestricted vegan diets can readily attain 0.7–0.9 g/kg/day of protein, enough for CKD or non-CKD populations [7]. Vegetarian patients on hemodiafiltration have also been able to attain even higher amounts of protein intake, estimated at 1.1 to 1.25 g/kg/day of protein, without any signs of malnutrition [8]. Vegetable-based diets are not only nutritionally adequate, but also have pleiotropic effects that may be beneficial for the treatment of CKD patients. In this review, we address the reasons why plant-based diets may be advantageous for renal patients (Figure 1). Obviously, in CKD patients the risk of hyperkalemia with these diets is an important limitation. Currently, there are no clinical studies that guarantee the safety of a diet richer in vegetables and fruits in this population [9].

## 2. Effects of Vegetable-Based Diets

**Figure 2:**
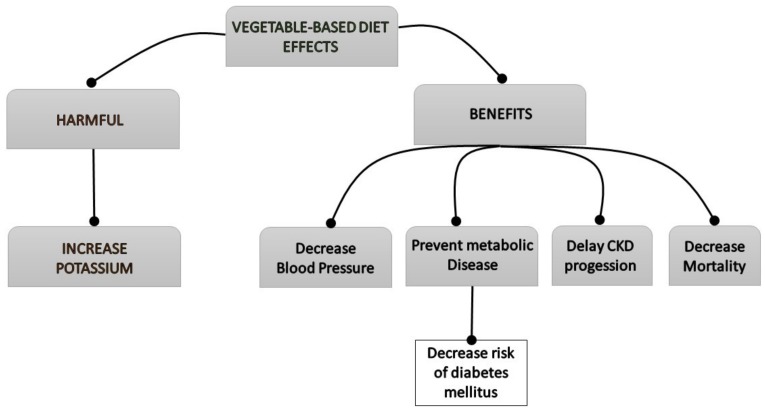
Flowchart of the clinical effects of the vegetable-based diet on the patient with chronic kidney disease (CKD).

Vegetable-based diets, though they show several beneficial effects on renal patients, can also favor some harmful events, such as hyperkalemia (Figure 2).

### 2.1. Vegetarian Diets and Gut Microbiota

A healthy gut microbiota is essential for the health and well-being of the host. In CKD, there is a dysbiotic gut microbiota characterized by a reduced diversity and an imbalance with a decrease in commensal bacteria and an increase in pathobionts and uremic toxins-producing bacteria [10,11]. Thus, restoring a healthy gut microbiota in uremic patients is an area of increasing research in nephrology. Dietary habits are the key modifiers of gut microbiota, depending on the duration of the diet and its nutritional composition [12]. The metabolism of colonic bacteria is regulated by the availability of nutrients and, specifically, the fiber content and the rate of dietary fiber vs. nitrogen [13]. A fiber-rich diet, such as a vegetarian/vegan diet, reduces protein fermentation, increases the carbohydrate fermentation [14], and may improve the dysbiosis associated with CKD by promoting the expansion of saccharolytic bacteria (bifidobacteria and lactobacilli) and the reduction in pathogenic bacteria species. A fiber-rich diet also increases the production of short-chain fatty acids (SCFA) (<6C), such as acetate, propionate, or butyrate by commensal bacteria that provide energy to the gut microbiota, allowing amino acids that reach the colon to be incorporated into the bacterial proteins and be excreted in feces, instead of being fermented to uremic toxins. Butyrate is taken up by colonocytes and used as their primary energy source [15]. SCFA also helps to maintain the functionality and integrity of the intestinal barrier, preserves the luminal pH, inhibits the growth of pathogens, and influences the intestinal motility [16,17]. In this sense, supplementation of hemodialysis (HD) patients with the SCFA propionate reduced proinflammatory parameters, oxidative stress, and the levels of some gut-derived uremic toxins; as well as improved the insulin resistance, iron metabolism, and quality of life, in a recent pilot study [18]. Dietary fiber, by increasing intestinal motility [19], reduces the time for fermentation of amino acids, improves the composition of the dysbiotic microflora, and enhances the excretion of human and bacterial byproducts, thus reducing the formation and/or accumulation of uremic toxins. Conversely, constipation, which is common among CKD patients, worsens the dysbiosis of gut microbiota, and contributes to the uremic status and to the risk of hyperkaliemia [20], and has been recently demonstrated to be a risk factor for the development and progression of CKD [21], likely due to the accumulation of uremic toxins and worsening of gut dysbiosis.

A previous study reported a significant decline in butyrate-producing bacteria in advanced kidney disease [22]. A more recent study, however, found a decrease in acetate, with no changes in propionate or butyrate levels, but an increase in valerate concentrations in advancing kidney disease. In this study, higher levels of valerate were associated with prevalent coronary artery disease. Although the mechanisms of this association remain speculative, the study provides evidence for impaired gut-microbiota-derived SCFAs with advancing CKD [23].

Dietary prescriptions in CKD (e.g., restriction of potassium-rich fruits and vegetables) results in a poor dietary fiber intake, slowing the intestinal transit time, and reducing the production of SCFA, which together with the formation of ammonium hydroxide increases the pH of the colonic milieu, further aggravating gut dysbiosis and favoring an efficient protein fermentation. In the absence of fermentable carbohydrates, protein fermentation induces the formation of potentially deleterious byproducts, such as sulfides, amines, ammonia, and phenols [24] that can accumulate in CKD and exert their harmful effects on the host [10]. In contrast, in vegan or vegetarian patients there is a reduced abundance of pathobionts and a greater abundance of symbionts [25]. 

However, it is unclear whether adhering to a vegetarian/vegan diet can promote a stable shift toward a healthier gut microbiota and maintain their long-term benefits in CKD. 

Among the compounds associated with vegetarian diets that have demonstrated salutary effects on the intestinal flora, several studies have shown the favorable effects of amylose, a vegetable unabsorbable complex carbohydrate that has been described to promote changes in the flora beneficial to the host both in CKD animal models [26] and end-stage renal disease (ESRD) patients [27] through the restoration of colonic epithelial barrier and attenuation of oxidative stress and inflammation [28], improvement of microbial dysbiosis in ESRD patients and retarding CKD progression. These compounds also improve the gut microbial dysbiosis and metabolomic profile [29] in CKD rats.

The fiber content of vegetarian/vegan diets is high and reaches the recommended levels of 20–30 g/day for CKD patients and 20–25 g/day in dialysis, according to the National Kidney Foundation (NKF) [30]. 

### 2.2. Vegetarian Diet and Metabolic Acidosis

Metabolic acidosis is a common complication of CKD resulting from the inability of the kidney to excrete the daily dietary acid load. Metabolic acidosis increases the risks of hypertension [31], heart failure [32], muscle wasting [33], bone loss, chronic inflammation, progression of renal failure, and death [34]. 

Current guidelines recommend treating metabolic acidosis in patients with CKD and serum bicarbonate <22 mEq/L [30]. Oral bicarbonate supplementation improved metabolic acidosis in individuals with CKD and slowed the rate of creatinine clearance decline from 5.93 to 1.88 mL/min/1.73 m^2^ per year [35]. 

Western diets are largely acid-producing since they are deficient in fruits and vegetables and rich in animal proteins [36]. Such diets can induce metabolic acidosis in individuals with reduced glomerular filtration rate (GFR), including otherwise healthy elderly persons [37], while proteins of animal origin (rich in sulfur-containing amino acids) increase the dietary acid load, worsening acidosis in CKD patients [38]. Foods such as meat, eggs, cheese, and grains increase the net acid load, whereas fruits and vegetables reduce it.

Metabolic acidosis is most common in patients with CKD stages 3b–5 [39]. Individuals with CKD stages 1–2 or 4 due to hypertensive nephropathy were placed on one year of dietary acid reduction with base-producing fruits and vegetables vs. oral sodium bicarbonate (NaHCO_3_). At the end of the study they had higher plasma bicarbonate and lower urinary indices of kidney injury than at baseline, consistent with improved metabolic acidosis and reduced kidney damage, and this base-producing diet appeared to be an effective kidney protective adjunct to blood pressure (BP) control with regimens that include angiotensin-converting enzyme (ACE) inhibition [40,41]. Thus, diets rich in vegetables and fruits might lower the dietary acid load and induce similar beneficial results as an alkali therapy in CKD patients.

### 2.3. Phosphorus and a Vegetarian Diet

Hyperphosphatemia is an independent risk factor for mortality in CKD patients. Hyperphosphatemia results from a positive phosphorus balance in renal patients, which results in a compensatory secondary hyperparathyroidism and an increase in fibroblast growth factor-23 levels (FGF-23) [42]. Typical western diets, which are usually rich in proteins, mostly from animal sources, are rich in phosphate. In addition, a nonnegligible amount of phosphorus is also provided by hidden preservatives or additives added to processed and fast foods [43]. 

The bioavailability of phosphorus varies widely according to sources. The intestinal absorption rate of phosphorus from animal sources reaches 80%, whereas intestinal absorption from a vegetarian source, which is mostly in the form of phytate, does not exceed 30% to 40% [44]. This is due to the fact that the accumulation of phosphorus in plants is in the form of phytates, which is necessary for their enzymatic hydrolysis by phytase; however, it is absent in mammals, which makes the released phosphorus available for absorption. Phosphorus from animal proteins is in the form of organic phosphate, which is readily hydrolyzed and absorbed [45]. In a crossover trial, Moe and colleagues compared the effects of isocaloric vegetarian and meat diets on phosphorus metabolism in 9 CKD (stage 3–4) patients [46], showing a significant reduction in serum phosphorus and FGF-23 levels and a decreased urinary 24-h phosphorus excretion in the vegetarian diet group compared to the meat-diet group. The authors concluded that vegetarian-based diets could be recommended for the control of phosphorus homeostasis in CKD patients. This is particularly relevant since increased FGF-23 levels appear to be independently associated with mortality among CKD patients [47]. 

### 2.4. Microbiota-Derived Uremic Toxins and a Vegetarian Diet

The gut microbiota also contributes to the generation of uremic toxins, such as indoxyl sulfate (IS), indole-3 acetic acid, *p*-cresyl sulfate (PCS), or trimethylamine *N*-oxide (TMAO), among others; which are produced by the breakdown of amino acids or amines, and are normally excreted by the kidneys, but accumulate in the presence of CKD, exerting their deleterious effects [10,48,49]. Some of them are protein-bound and their elimination through dialysis is scarce [48]. IS and PCS have been involved in the increased cardiovascular risk, contributing to the inflammation and oxidative stress, as well as insulin resistance, or CKD progression, and are independently associated with mortality in CKD [50]. TMAO is a proatherogenic byproduct of the gut microbiota, which has also been associated with increased mortality in CKD [51].

Urea is also emerging as a real uremic toxin. In CKD there is an enhanced influx of urea into the intestinal lumen via the entero-hepatic cycle, where it is converted to ammonia and ammonium hydroxide by the gut bacteria, which is enhanced by the abundance of urease-producing bacteria in these patients [52]. Ammonia induces the depletion of key proteins of the colonic tight junctions, which is associated with the disruption of the gut epithelial barrier, favoring endotoxemia and bacterial translocation to the circulation, contributing to the systemic inflammation in CKD. Ammonia also increases the colonic pH, further worsening the uremic dysbiosis. Urea also promotes insulin resistance and beta-cell dysfunction. In addition, the high levels of urea in CKD also cause an enhanced production of the breakdown product cyanate, which induces protein carbamylation, a posttranslational modification that alters the structure and function of the proteins. Carbamylation has been associated with renal fibrosis, atherosclerosis, and anemia [53]. Hyperuricemia is also common in CKD and has been associated with several comorbidities, such as CKD, obesity, hypertension, diabetes, or cardiovascular disease [54].

The production of the aforementioned uremic toxins can be influenced by dietary changes. The uremic dysbiosis may be further aggravated by the dietary restrictions in CKD patients, in order to limit the intake of potassium-rich fruit and vegetables (fiber) and phosphorus-rich dairy products, such as yogurt and cheese (probiotics), to prevent hyperkalemia and hyperphosphatemia, respectively [55].

In CKD patients, there is a direct relationship between the protein/dietary fiber ratio and the PCS and IS levels. Therefore a diet with a lower protein/fiber index, such as a vegetarian/vegan diet, could reduce the levels of these uremic toxins [13]. In this sense, in subjects with a normal renal function, a vegetarian diet reduces the urinary excretion of PCS and IS by approximately 60%, reflecting their decreased generation [56]. A vegetable-based very low protein diet supplemented with keto-analogues also reduces uremic toxin levels [57]. In dialysis patients, a vegetarian diet was associated with lower serum blood urea nitrogen, creatinine, uric acid, or C-reactive protein, while serum potassium, albumin, prealbumin, muscle strength, subjective global assessment, and activities of daily living showed no differences with nonvegetarian patients [58]. In vegetarian patients on maintenance hemodiafiltration, plasma levels of IS and PCS were lower compared to the patients on an omnivore diet; they also showed lower urea and phosphate levels [8].

A diet rich in fiber reduces urea levels [19,59,60] and has been associated with a significantly lower risk for hyperuricemia and its comorbidities, likely due to a suppression of the digestion and/or absorption of dietary purines [19,54]. Furthermore, a diet rich in fiber reduces serum levels of other uremic toxins, specially PCS in several studies and a meta-analysis [19,61,62,63]. Studies in non-CKD individuals have observed that consumption of resistant starch decreased fecal phenol and ammonia [64], while fermentable carbohydrates increases fecal nitrogen and reduces urinary nitrogen excretion in non-CKD [65] and CKD patients [66].

Vegetarian diets have lower contents of lecithin, choline, and l-carnitine, which might result in a lower production of TMAO. A recent study of non-CKD populations revealed that TMAO production was mainly associated with the ingestion of red meat (rich in L-carnitine), while L-carnitine enhanced TMAO production in omnivores but not in vegetarians [67,68]. Vegetarian diets are rich in polyphenols and antioxidants; and in a recent study resveratrol, a natural polyphenol present in grapes and berries, decreased TMAO levels and attenuated TMAO-induced atherosclerosis in an animal model by modifying the gut microbiota [69]. 

### 2.5. Magnesium and a Vegetarian Diet

Magnesium is a divalent cation essential for human health. It plays a role in a number of physiological processes, such as a cofactor in enzymatic reactions, regulates transmembrane transport of other ions, and has structural functions [70]. Hypomagnesemia is a common finding in CKD and end-stage renal disease (ESRD) patients, which can be due to dietary restrictions, use of certain drugs (e.g., proton-pump inhibitors [71]), proteinuria-induced tubular damage [72], or to a low magnesium content in the dialysate. Magnesium has been shown to have beneficial effects on cardiac function and vascular calcification [73], the last by directly inhibiting hydroxyapatite crystal formation [74], an effect which may be especially relevant in the presence of the procalcifying milieu in CKD [75]. In experimental studies, magnesium has also demonstrated its protective effect against endothelial cell dysfunction and oxidative stress [76]. In cohort studies in HD patients, peritoneal dialysis and CKD patients, lower serum magnesium levels have been associated with an increased risk of all-cause and/or cardiovascular mortality, even after adjusting for other factors [77,78,79,80]. Further, magnesium inhibits hyperphosphatemia-induced vascular calcification, and a recent study observed that the risk of hyperphosphatemia-associated cardiovascular death was attenuated with increasing serum magnesium levels [81]. Hypomagnesemia has also been associated with an increased incidence of CKD [82] and a faster progression of the disease [83]. In small studies in dialysis patients or CKD patients, magnesium supplementation has been associated with a decreased intima-media thickness [84], vascular calcification progression [85], or improved serum calcification propensity [86]. On the other hand, because of the renal excretion of Mg, there is a potential risk that a diet rich in magnesium or magnesium supplements might result in toxic hypermagnesemia in patients with advanced CKD, although the evidence from small studies does not support this setting [86].

Regarding the magnesium content in the diet, it is abundantly found in green leafy vegetables, beans, legumes, and nuts, while processed foods are poor in this cation. In CKD or ESRD, the usual dietary restriction of potassium may also lead to a low magnesium intake. In contrast, a vegetarian diet meets the daily nutrient requirement for HD patients according to the NKF [30].

### 2.6. Vitamin K and a Vegetarian Diet

Vitamin K refers to a group of fat-soluble vitamins that are cofactors for γ-glutamyl carboxylase, the enzyme that activates several vitamin K-dependent proteins involved in coagulation, bone formation, and inhibition of vascular calcification. Since vitamin K is involved in the carboxylation of matrix γ-carboxyglutamate (GLA) protein (MGP) and bone GLA protein (BGP or osteocalcin), vitamin K deficiency is associated with an increased risk of vascular calcification and bone demineralization, which may be especially relevant in the CKD population [87].

The main dietary sources of vitamin K1 are green vegetables, especially spinach, broccoli, kale, and brussels sprouts. Vitamin K2 is predominantly found in fermented foods, such as cheese, curd, and natto. While vitamin K1 is important for the hepatic gamma carboxylation of clotting factors, vitamin K2 is important for the extrahepatic carboxylation of proteins, such as MGP or osteocalcin. 

Several studies have evaluated the dietary intake of vitamin K in dialysis and CKD patients, and found that the usual CKD diet is deficient in vitamin K [88,89] in hemodialysis patients, and in CKD patients in some [90], but not all studies [91]. Dietary restrictions in CKD patients, such as low-potassium (poorer in green leafy vegetables rich in K1) and low-phosphate diets (lower consumption of dairy products rich in K2) can favor this deficiency. However, the vitamin K status depends not only on diet, but also on gut bacterial synthesis, the endogenous recycling and drug interferences (e.g., warfarin, phosphate binders). A deficient production of vitamin K by gut microbiota due to the uremic dysbiosis could also play a role in this deficiency [92]. In some cases, the deficient vitamin K status in CKD might also be due to its depletion due to its high requirements by vitamin K-dependent proteins to inhibit calcification [93]. Statins, commonly prescribed in this high cardiovascular risk population, may further aggravate vascular calcification in CKD by inhibiting the synthesis of vitamin K2 from vitamin K1 in vascular smooth muscle cells [94]; while the prescription of vitamin K antagonists for the prevention of thromboembolic events in atrial fibrillation, a common arrhythmia in this population, further aggravate vascular calcification and arterial stiffness [95]. A deficiency in vitamin K2 can be especially relevant in CKD patients who are prone to vascular calcification and osteoporosis. In fact, plasma levels of dephosphorylated uncarboxylated MGP (dp-ucMGP), as a marker of vitamin K deficiency in CKD, is associated with cardiovascular disease and with overall survival [96], as well as with low bone mineral density and increased fracture risk [97].

In a small study, vitamin K2 supplementation in CKD stages 3–5 patients reduced the progression of atherosclerosis and significantly changed the levels of calcification promoters and inhibitors: dp-ucMGP, osteocalcin, and osteoprotegerin [98]. A vegetarian diet or a very low protein diet (plant-based) supplemented with keto-analogues are richer in vitamin K1 [91].

### 2.7. Effects of a Vegetarian Diet on Inflammation and Oxidative Stress

Low-grade inflammation and oxidative stress are common findings in CKD, and have been associated with the progression of renal dysfunction, as well as other complications of CKD, such as atherosclerosis, cardiovascular risk, or protein-energy wasting [99]. 

As previously mentioned, the dysbiotic gut microbiome in CKD favoring pathobionts overgrowth, together with the increased intestinal barrier permeability, contributes to the systemic inflammation and oxidative stress in CKD patients through the translocation of bacteria and bacterial products into the systemic circulation [100,101].

The type of diet is key to the modulation of inflammation. Diets rich in fruits and vegetables, vitamins, and antioxidants have been associated with lower levels of inflammatory markers [102]. In contrast, a western diet (rich in animal proteins and fats) stimulates the overgrowth of proteolytic bacteria, which results in dysbiosis, the accumulation of proteolytic-derived uremic toxins, and may promote CKD progression. 

In experimental studies, a diet rich in undigestible fiber improves the markers of oxidative stress, and reduces inflammation and kidney damage in CKD rats [103]. Similar results have been reported in CKD patients [104,105]. Vegetarian diets have been associated with a reduction in inflammation [106]. A diet rich in fiber improves the uremic dysbiosis with a decrease in pathobionts and an increase in commensal bacteria, and enhances the production of SCFA that help to maintain the intestinal barrier functionality and integrity by inducing intestinal epithelial cell secretion of IL-18, antimicrobial peptides, mucin, and up regulating the expression of the tight junctions. SCFA are involved in immune system activation by inducing neutrophil chemotaxis and enhancing their phagocytosis and through regulation of T cell function, both through the regulation of dendritic cells and the proliferation of regulatory T lymphocytes (Tregs) [107]. 

The high prevalence of oxidative stress observed in CKD patients is due to an imbalance between the increased production of reactive oxygen species and a low antioxidant status, in part due to the low intake of antioxidants from the usual diet. Oxidative stress has been associated with cardiovascular risk in CKD and may influence the progression of renal injury [108]. Long-term vegetarian diets have been associated with reduced markers of oxidative stress as compared with omnivore diets [109]. This may be due to the increased fiber intake, low-saturated fatty acid content, higher content of antioxidant vitamins, as wells as polyphenols and flavonoids, which are potent antioxidants. In a recent study by our group on an in vitro model of uremic endothelial dysfunction, we reported the beneficial effects of these polyphenols and flavonoids [110].

### 2.8. Vegetarian-Diet and Intestinal Motility

Cereal fibers are known to increase fecal weight and accelerate transit time, but fewer data are available on the effects of fruits and vegetable fibers on bowel regularity. Both fecal weight and transit time are the key indicators of digestive health [111]. Dietary fiber plays an important role in the adequate functioning of the gastrointestinal tract and has been advocated to improve the intestinal function [112,113]. European Nutrition guidelines call for consumers to meet their daily dietary fiber intake goal by eating a variety of fruit and vegetables and whole grains [113]. 

de Vries et al. [111] observed that nonfermentable dietary fibers from cereals and vegetables contribute more to fecal weight than fermentable fibers from fruits. For patients with transit time higher than 48 h, the transit time was significantly reduced with fibers from cereals and vegetables, regardless of their fermentability. This review showed that the estimated fermentability determined the role of fiber in total fecal weight. In healthy individuals, a normal physiological transit time varies between 40 and 60 h [114]. 

Risk of many chronic disorders (e.g., cancer, diabetes, or cardiovascular diseases) may be reduced by the regular consumption of fruits and vegetables or other plant-based foods [115,116]. Further, a diet rich in fruits, vegetables, and fiber could reduce the mortality in the general population [117]. 

In addition to the above benefits, a number of bioactive compounds naturally present in fruits and vegetables have antioxidant and anti-inflammatory effects. The combinations of different pure bioactive compounds or their extracts from food sources can have synergistic benefits conferred by individual bioactive compounds. However, concurrently consumed bioactive compounds may affect the intestinal absorption of each other. The interactions of phytochemicals may enhance or reduce the bioavailability of a given compound, depending on the facilitation/competition for cellular uptake and transportation taking place between them [118,119], e.g., for some vegetable combinations, such as tomato and onion, or tomato and lettuce, a synergic antioxidant activity is observed in the raw and digested extracts, but not in the absorbed extract. Only the combination of tomato and garlic shows synergistic bioactivities in all forms tested (raw, digested, and absorbed), probably because both products contain highly bioactive and bioavailable active constituents. A number of phytochemical mixtures and food combinations have been reported to provide synergistic antioxidant, anti-inflammatory or inhibitory effects on cancer cell proliferation [120].

The adoption of a plant-based diet, such as vegan or a plant-based very low protein diet, has positive effects on the bowel status, reducing the microbial metabolites originated by protein intestinal fermentation.

### 2.9. Risk of Hyperkaliemia

Hyperkalemia is a common electrolyte abnormality in CKD patients. The incidence of hyperkalemia increases as GFR declines and has been found to be as high as 31% among patients with an estimated glomerular filtration rate (eGFR) ≤ 20 mL/min/1.73 m^2^ [121]. To prevent hyperkaliemia, CKD patients are advised to reduce their potassium intake to 2000–3000 mg/day (50–75 mEq/day), which is significantly lower than the potassium recommendations of 4700 mg/day, according to the recent US Department of Agriculture guidelines. Thus, dietary recommendations to CKD patients restrict plant-based foods, such as seeds, nuts, beans, and peas, as well as fruits and vegetables. The potential health benefits of plant-based/high-potassium diets may be related in part to their alkalinizing effects. The alkalinizing effects of potassium-rich plant foods may explain the reductions in metabolic acidosis in nondiabetic CKD patients who were acidemic [40]. Potassium derived from plants may promote intracellular potassium distribution, as well as promote its fecal excretion due to the natural fibers found in plant-based diets [20,122].

No studies have shown differences in serum potassium levels in patients consuming predominantly plant-derived versus omnivore-derived potassium sources. Two trials conducted by Goraya et al. [41,123] including CKD patients stages 2 and 3, found that reducing the dietary acid load with a diet rich in fruits and vegetables did not induce hyperkalemia in their patients, while correcting metabolic acidosis similarly to bicarbonate supplementation and reducing markers of kidney injury. Although the study excluded individuals at high risk for hyperkalemia, such as those with diabetes or those with potassium levels >4.6 mEq/L.

Thus, caution is needed in patients with eGFR < 30 mL/min/1.73 m^2^, for whom restricting dietary potassium can be advisable. Plant-based diets, despite their relatively higher potassium contents, have not shown to induce hyperkalemia in these patients [124]. Further, the use of cooking techniques that reduce K and P content from vegetables and legumes, can reduce the risk of hyperkalemia and will be considered later.

## 3. Effect of Vegetable-Based Diets on Renal Patient Complications

### 3.1. Vegetarian Diet in Hypertension and Diabetes Mellitus

Many studies recommend plant-based diets, such as the Dietary Approaches to Stop Hypertension (DASH) diet or the Mediterranean diet (MD), which are rich in fiber; are low in saturated fat and processed meats; contain sources of potassium, phosphorus, magnesium, and calcium; and have a low sodium content. MD and DASH diets are similar, both have been associated with a lower risk of mortality from cardiovascular disease and slow the progression of kidney disease [125], and have been recommended for primary and secondary cardiovascular disease prevention. Emerging evidence in patients with CKD suggests that these diets may be helpful to delay progression and prevent metabolic complications [126]. Reluctance to recommend a MD to the CKD patient may arise when some of the typical components of the MD pyramid are in conflict with the traditional dietary restrictions of CKD.

ESRD patients treated with hemodialysis, experience an annual mortality rate of 10–20%, largely due to cardiovascular causes, and they do not share the benefits from interventions of proven benefit in the general population (e.g., Statins, ACE inhibitors, etc) to date. MD and DASH diets are associated with reduced mortality in the general population, but their effects in patients on HD are uncertain. In a cohort study involving 9757 patients on HD followed for 2.7 years, there was no association between these dietary patterns and cardiovascular or all-cause mortality. These findings suggest that diets that are protective in the general population may not predict better cardiovascular outcomes in patients on HD, despite the potential benefits in this population [125,127], although randomized controlled trials are needed to appropriately answer this question. 

Numerous cross-sectional studies have found that, in developed countries, blood pressure (BP) was lower among vegetarians than in nonvegetarians, after adjustments for age, sex, and body weight, although the increase in BP with age is also observed in this population [128]. Vegetarian diets have beneficial effects on weight control, while lower rates of overweight and obesity among vegetarians have been confirmed in different series [129]. In an analysis of three prospective cohort studies including >120,000 men and women, investigating the relationship between lifestyle factors and weight changes at 4-year intervals, consumption of plant-based foods was inversely associated with weight gain [130]. In a recent meta-analysis, including 15 intervention trials, prescription of a vegetarian diet of >4-weeks duration, without energy intake restrictions, was associated with a decrease of 3.4 Kg of body weight [131]. A recent excellent review on the influence of a vegetarian diet in CKD [132] concisely analyzes the dietary components capable of lowering BP including a reduction of sodium or protein intake, and a higher intake of potassium, complex carbohydrates, and dietary fiber. 

When comparing ovo-lacto-vegetarians with omnivores in a population of diabetic patients, the first group showed higher insulin sensitivity than their omnivorous counterparts and the degree of insulin sensitivity was correlated with years of vegetarian diet. [133]. Whole-grain products and vegetables generally have low glycaemic index values, and individuals following vegetarian diets are less than half as likely to develop diabetes compared to nonvegetarians [134]. After adjustment for several covariates (age and lifestyle) the prevalence of type 2 diabetes mellitus (DM2) increased from 2.9% in vegans to 7.6% in nonvegetarians, while the prevalence was variable in participants consuming lacto-ovo-, pesco-, and semi-vegetarian diets [135]. 

A systematic review and meta-analysis including 255 DM2 patients (17 lacto-ovo-vegetarians and 238 vegans) showed that consumption of a vegetarian diet, combined with exercise, was associated with a dramatic reduction in the use of glucose-lowering medications and in hemoglobin A1c, as well as a nonsignificant reduction in fasting plasma glucose concentration [136].

Finally, proteins from vegetables have a lower impact on renal haemodynamics than animal-based proteins. Replacing proteins of animal origin with vegetable-based proteins may decrease renal hyperfiltration, proteinuria and, hypothetically, in the long-term, the risk of developing renal failure [136,137].

### 3.2. Progression of Chronic Kidney Disease

**Figure 3:**
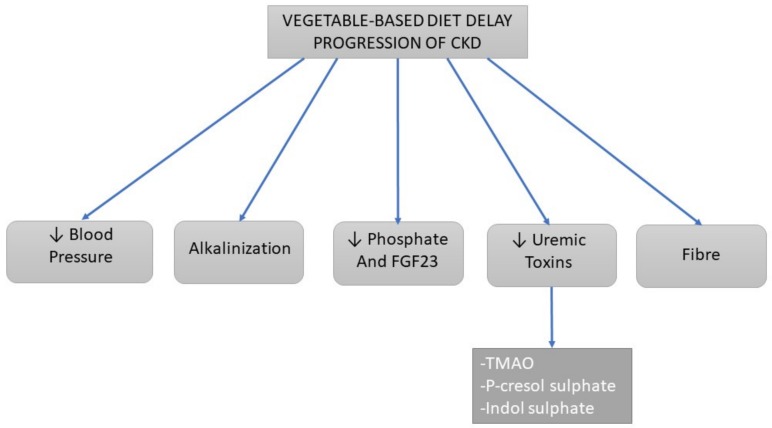
Scheme of the actions of the vegetable-based diet on the progression of kidney damage in patients with CKD according to the literature. Legend: ↓ Decrease.

Previous studies suggest that consumption of fruits and vegetables by reducing the dietary acid load and improving metabolic acidosis may slow the reduction of eGFR in patients with CKD [40] (Figure 3). As mentioned above, diets rich in vegetables and an increase in total fiber intake are associated with reductions in the progression of CKD, attributed to several causes: reduced consumption of nutrients such as protein, sodium, or acids, increased potassium over sodium intake, decreased phosphorus load, and increased intake of fiber, antioxidants, vitamins, and chemicals such as sulforaphane that have been linked to improved outcomes in patients with CKD [38]. 

#### 3.2.1. Alkalinizing Effects

Metabolic acidosis increases the production of endothelin [138] and angiotensin II by the kidney, with the subsequent stimulation of aldosterone, with an adaptive increase in ammonia production by the remaining renal tubules, which in consequence activates complement and the inflammatory cascade, together promoting renal fibrosis and a decline in GFR [139]. Scialla et al. [140] has described a direct association between the dietary acid load and a decrease in GFR rate. CKD patients, with the reduction of the nephron mass, have an increase in renal-mediated ammoniogenesis and the distal excretion of acid, mediated by Renin-Angiotensin-Aldosterone System (RAAS) activation and endothelin-1, resulting in renal damage [141,142,143]. In a high-risk African American population, a high dietary acid load was independently associated with an increased likelihood of albuminuria and reduced renal function [144]. Acid-producing diets led to endothelin-mediated GFR decline, and correction of acidosis with oral alkali slows GFR decline, preserves glomerular filtration, and reduces renal endothelin production in rats with reduced nephron mass [138]. Dietary acid-induced kidney injury in rats with intact or reduced nephron mass is due to tubulointerstitial injury, mediated through endothelin receptors. Studies support the fact that renal injury increases with dietary acid load, while correction of acidosis with oral NaHCO_3_ or base-rich vegetable diet reduced renal injury in subjects with CKD due to hypertensive nephropathy [41,123]. 

#### 3.2.2. Dietary Sodium and Blood Pressure

A vegetable-based diet, but not NaHCO_3_, reduced the systolic blood pressure in CKD patients, suggesting a possible advantage of this diet poorer in sodium over alkali administration as a strategy to reduce dietary acid levels for kidney protection. These results encourage long-term studies to determine whether a vegetable-based diet, similar to NaHCO3, is an effective addition to blood pressure reduction and inhibition of ACE to slow the decline of GFR in hypertensive nephropathies as well as in other nephropathies [41].

Lin et al. showed that the DASH diet was associated with a 45% reduced risk of decreased renal function (decreased GFR ≤ 30% from baseline), while the western diet was associated with higher risk of decreased renal function [145]. 

Fruits and vegetables are a main source of fiber, potassium, and nitrate. Studies have reported that dietary potassium by reducing blood pressure [146] and dietary fiber through decreasing inflammatory markers and blood pressure may protect against CKD [147]. Vegetables, especially leafy greens, are rich sources of nitrates, and the nephroprotective effect of these foods is related to their nitrate content [148]. Although the main source of nitric oxide (NO) is generated from L-arginine, recent research indicates that NO is also generated by the nitrate–nitrite–NO pathway from dietary nitrate or nitrate supplementation [149].

#### 3.2.3. Uremic Toxins

A diet with a higher vegetable content further modifies the intestinal microbiota, which may result in increased production of SCFA [150] and a lower production of nephrotoxic uremic toxins [151]. Plant-based diets and dietary protein/fiber index were significantly associated with serum IS and PCS levels [56], beyond its well-known association with kidney function [13]. Dietary fiber may reduce the production of these uremic toxins by limiting proteolytic bacterial fermentation. Dietary modifications toward a lower protein-fiber index may contribute to lowering IS and PCS [13].

#### 3.2.4. Phosphate

In patients with advanced CKD, hyperphosphatemia has been associated with more rapid progression to ESRD [152]. High phosphate intake increases the phosphate burden and the resulting phosphaturia produces secondary kidney damage by inducing tubular injury and interstitial fibrosis [153]. Experimental studies showed that excessive phosphaturia was associated with renal injury, inflammation, oxidative stress, and decreases in renal Klotho [154], while phosphaturia has been associated with a deterioration in the renal function in CKD patients.

Intestinal absorption of phosphate from a vegetarian source, which is mostly in the form of phytate, does not exceed 30% to 40% [44] in contrast to the higher absorption of animal-based proteins. Thus, vegetarian-based diets may be recommended for the control of phosphorus homeostasis in CKD patients, and it could have an influence in reducing progression of CKD.

#### 3.2.5. Fiber Content

The Tehran Lipid and Glucose Study (TLGS) showed that plant-based diets rich in fiber were associated with a lower risk of incident CKD [155], while Mirmiram et al. observed inverse associations between total fiber intake and risk of incident CKD [156], suggesting that a high fiber intake, mainly from legumes and vegetables, may reduce the occurrence of CKD. However, the meta-analysis of Kelly et al. showed that there is limited evidence in the literature for the association of dietary fiber content with a reduced risk of ESRD, although cohort studies suggest that healthy dietary patterns, rich in fruit and vegetables, may lower the risk of progression to CKD, and decrease albuminuria and BP [126].

### 3.3. Mortality

Vegetarian diets are associated with lower all-cause mortality and with some reductions in cause-specific mortality. Vegetarians had 0.88 times the risk of all-cause mortality [157]. In the European Prospective Investigation into Cancer and Nutrition (EPIC)–Oxford study, that included subjects with normal renal function, found that consuming a vegetarian diet was associated with lower risk of ischemic heart disease, a finding that was probably mediated by differences in non-HDL cholesterol, and systolic blood pressure [2]. Moreover, in a prospective cohort study by the US health care professionals that included 131,342 participants from the nurses and health professionals follow-up study with a follow-up of more than 25 years, showed that a high animal protein intake was positively associated with cardiovascular mortality, while a higher plant-based protein intake was inversely associated with all-cause and cardiovascular mortality [158]. Finally, the meta-analysis of Kelly et al. has shown that a healthy dietary pattern, including increased fruit and vegetable, fish, legume, whole grains, and fiber intake, and reduced red meat, sodium, and refined sugar intake, is associated with lower mortality in CKD patients [126].

Given the total protein intake, a higher proportion of protein from plant sources is associated with lower mortality in CKD. There are several reasons because of which a high plant-based protein intake decrease mortality. Protein intake from plants is associated with lower production of uremic toxins, and lower serum phosphorus levels. This diet also has a significant influence on the cholesterol metabolism. The analysis of data from the prospective longitudinal Chronic Renal Insufficiency Standards Implementation Study (CRISIS) revealed that higher serum phosphate, even within the normal range, was associated with an increased mortality in patients with CKD stages 3–4 [159]. Isakova et al. [160] suggested that dietary phosphate restriction in combination with phosphate binder therapy may play a role in reducing FGF-23 levels in patients with CKD stages 3–4 and normal serum phosphate levels, since FGF-23 is recognized as a novel risk factor for ESRD, cardiovascular disease, and mortality.

Therefore, a higher proportion of dietary protein from plant sources might be associated with lower mortality in CKD [161]. Despite plentiful evidence from observational studies regarding the benefit of plant-based proteins in reducing cancer risk [162], cardiovascular mortality [163], blood pressure [164], and diabetes [165], there is no clear evidence for renal disease.

As previously mentioned, metabolic acidosis accompanying CKD increases mortality and contributes to morbidity, such as decreased bone mineral content, increased protein catabolism, and possibly kidney disease progression. The most effective treatment strategy available to clinicians is dietary H^+^ reduction that can be accomplished with Na^+^-based alkali and/or with base-rich foods, like fruits and vegetables [166]. In the African American Study of Kidney Disease and Hypertension (AASK) cohort of more advanced CKD, a diet high in animal sources of protein may lead to higher estimated net endogenous acid production, which was associated with decreased serum bicarbonate levels [167], and each 1-mmol/L increase in serum bicarbonate level within the normal range was associated with decreased risk for a composite of death [168].

## 4. Practical Tips

### 4.1. Role of the Mediterranean Diet

The traditional MD is characterized by a high intake of olive oil, fruit, nuts, vegetables, and cereals; a moderate intake of fish and poultry; a low intake of dairy products, red meat, processed meats, and sweets; and moderate consumption of wine with meals (Table 1) [137]. Emerging evidence in patients with CKD suggests that these diets may be helpful to delay progression and prevent complications [169]. The reluctance to recommend an MD to the CKD patient may arise when some of the typical components of the MD pyramid conflict with the traditional dietary restrictions of CKD (Table 2). The European Renal Nutrition (ERN) working group of the European Renal Association–European Dialysis Transplant Association (ERA-EDTA) aims to summarize arguments in favor of and against adopting the MD as a healthy dietary pattern and lifestyle for the CKD population [126].

Some reports establish that food plays a central role in the social and cultural life of the Mediterranean area and qualitative elements such as cooking, physical and social activities, outdoor life, and adequate rest strengthen the healthy effects of MD [126,169]. 

Protein intake in the MD aligns with a controlled protein intake for CKD (0.8 g/kg/day). Another interesting aspect is the source of protein, which in the MD comes predominantly from vegetables, fish, and white meat. Red meat and processed meats are less often consumed, which may convey a lower amount of dietary sodium, phosphate, and potassium. Such habits have been associated with lower cardiovascular and cancer risk in the community, but also with lower risk of incident CKD and of ESRD in individuals with normal kidney function [126].

In CKD patients, the benefits of plant-based versus animal-based protein have been poorly studied. Old studies addressing the impact of acute protein loading (short-term interventions of 4–12 weeks) showed no or mixed effects of protein sources on changes of eGFR [126]. Two more recent short-term randomized controlled trials in patients with CKD stages 3–5 showed that adherence to a plant-based diet as compared with a meat-based diet was effective in maintaining serum phosphate targets and reducing FGF-23 [46,170]. 

A typical MD provides 50% of the lipid-derived energy from monounsaturated fatty acids (MUFA), 25% from polyunsaturated fatty acids (PUFA), and 25% from saturated fatty acids (SFA). Oleic acid is the main MUFA and is present in extra-virgin olive oil, which is also rich in polyphenols and vitamin E, and collectively has additive anti-inflammatory, antioxidant, and vasculo-protective properties [169]. Increased olive oil consumption has been consistently associated with a lower risk of all-cause mortality, CV mortality and morbidity, and stroke in the general population and in individuals with established CVD. Such reductions are not always observed in other studies where other sources of MUFA of both animal and plant origin have been used. [126]. The MD is also rich in n-3 PUFAs (both from the marine origin and from plants), which have triglyceride-reducing, anti-inflammatory, and anti-thrombotic properties [171].

The traditional MD is associated with traditional, local and eco-friendly products, and a low consumption of processed foods. The impact of the MD is therefore not only explained by its specific nutrients and foods, but also by the way these foods are produced, cooked, and eaten. However, the frozen products, particularly vegetables and legumes, maintain the food properties with lower phosphorous and potassium contents. 

The MD provides 30–50 g/day of fiber with a 1:1 ratio of soluble to insoluble fibers. Dietary fiber has important health-promoting properties. Besides its well-known benefits on the gastrointestinal health, individuals with higher intakes of dietary fiber appear to be at significantly lower risk for developing coronary heart disease, stroke, hypertension, diabetes, obesity, and certain gastrointestinal diseases. Increasing fiber intake lowers blood pressure and serum cholesterol levels, improves glycaemia and insulin sensitivity, and reduces inflammation [172]. The MD, with its abundant supply of high-quality complex carbohydrates (>50% whole grains) and dietary fiber, has a lower glycaemic index and increases the plasma levels of the anti-inflammatory adipokine, adiponectin. The Third National Health and Nutrition Examination Survey (NHANES III) study showed that fiber intake was low in most individuals (14.5 g fiber/day compared to the daily recommendations of 25 g/day), and also showed that a high fiber intake showed strong inverse associations with inflammation and mortality in the subset of patients with CKD. Specifically, each 10 g/day increase in total dietary fiber intake was associated with a 17% lower mortality risk [105]. The beneficial effect of dietary fiber may also be linked to the shift of gut microbial activity from a proteolytic toward a saccharolytic fermentation pathway, as mentioned above. It has been proposed that an MD style diet in combination with probiotic/prebiotic formulations, could be a valid therapeutic approach for CKD patients [173,174].

MD produces a basic net balance and has the potential to decrease the dietary acid load (DAL) and prevent the low-grade subclinical metabolic acidosis that is a feature of our modern western diets. Western diets, with a higher ratio of foods of animal origin compared to fruits and vegetable products, result in a higher acid precursor content in the body [175]. The possible benefits of a low DAL in healthy individuals with normal renal function come from observational studies, suggest potential benefits in reducing the incidence of diabetes, fractures, hypertension, CVD, or mortality. 

### 4.2. Practical Cooking Counselling

CKD patients have dietary restrictions in fruits and vegetables but, if cooked with the appropriate technique, its mineral content can be reduced. These restrictions are responsible for the noncompliance of the patients with the dietary recommendations since they are pushed to introduce completely new food habits [176]. 

Cooking techniques can promote the safe intake of legumes and vegetables, while avoiding hyperphosphatemia or hiperkalemia, but with these techniques also some healthy and essential nutrients can be lost, such as Mg and Zn. Oral supplements can be required, when necessary, to avoid these deficiencies [177]. Phosphorus, potassium, and sodium are the three most difficult minerals to control when kidney function is severely compromised. However, the amount in foods can be reduced by up to 80% owing to some cooking techniques, especially blanching and boiling. Using frozen or canned foods plus washing can also flush them out in the same proportion. Therefore, a healthy diet that ensures the intake of essential amino acids, mineral-rich fruits, vegetables, legumes, and dairy products, can be maintained keeping the intake of these three elements in a safe range with appropriate dietary counseling. Although there is no consensus on the maximum amount of K and P per serving of food in each stage of CKD, there are foods, such as raw legumes, whose content in these minerals is so high that they can only be consumed after following certain preparation recommendations. 

Professionals involved in the care of patients with CKD, at any stage or treatment modality, should promote increased intake of legumes and vegetables always using appropriate cooking techniques. In order to achieve these objectives, multidisciplinary teams consisting of nephrologists, renal nurses and renal nutritionists are needed.

### 4.3. Potential Risks of a Vegan Diet in CKD

A strict vegetarian diet can be poor in long-chain n-3 fatty acids, zinc, iron, and vitamin B_12_. Although some studies indicate a higher risk of osteoporosis in vegetarians than in the general population, differences in bone mineral density between vegetarians and the general population are not clinically relevant [178]. However, osteoporosis and bone-mineral disorders are common among CKD patients and the effects of a vegan diet on the risk of fractures should be evaluated in this population. Vitamin B_12_ should be monitored and supplemented in these patients if needed. Although the iron content in vegetarian and nonvegetarian diets are similar, the bioavailability of iron from plants is lower (low heme iron content or scarcely bioavailable iron, e.g., chelated by phytates) [179]. This can be relevant in CKD patients who have increased hepcidin levels, impairing iron absorption. Similarly, a diet without an animal source of food can be poor in zinc, as this element is less abundant in plant-based food than in meats, and poorly absorbed because of the presence of fibers and phytate. A vegetarian diet can also be poor in vitamin D, which needs to be monitored and supplemented as needed [179]. A vegetarian diet should also be controlled to ensure an adequate consumption of all essential amino acids by a careful combination of legumes and cereals. A diet relatively poor in n-3 fatty acids can be offset by increasing the intake of walnuts or flax seeds.

In summary, when balancing the evidence of the benefits and risk/harms of a vegetarian diet in CKD patients, a plant-based diet appears to offer important advantages to these patients (Table 2); and with good nutritional advice a plant-based diet will be safe. However, it must be recognized that a plant-based diet may pose significant restrictions, and may be difficult to follow by the patients. It is time to individualize/liberalize the diet for our CKD patients by offering counseling on diet richer in plant-derived foods, such a vegetarian, MD or DASH diets with careful monitoring of potential risks (such as hyperkaliemia). Further, new studies with large sample sizes and in the different stages of CKD or ESRD are needed to confirm these potential benefits and the safety of these diets. To achieve these goals involvement of multidisciplinary teams of nephrologists, nephrology nurses, and dietitians who can ensure an adequate and safe nutrition to renal patients is mandatory.

## Figures and Tables

**Table 1:** Main points of adherence to a Mediterranean diet (MD). In order to avoid the harmful effects of hyperkalemia, emphasis is given to the need to adapt cooking techniques.

Mediterranean Diet Characteristics
1.-Fruits and vegetables in every meal day
2.-Dairy products, preferably low fat: Every day
3.-Bread, Pasta or Rice: Every day
4.-Cereals and olive oils: Every day
5.-Nuts and olives: Every day
6.-Potatoes, White meat, Fish, Legumes and Eggs: Every week
7.-Reduced: Sweets, red and processed meat.
Biodiversity, fresh, seasonal, unprocessed and traditional culinary activity.

**Table 2:** Main studies of vegetable-based dietary interventions on clinical and biochemical parameters in renal disease.

Authors	Population	Dietary Intervention	Outcomes and Measurements	Reference
Barsotti et al.	22 stage III/IV CKD patients	Special vegan diet (SVD) vs. Conventional low-peotein diet (CLPD) vsunrestricted protein diet (UPD)	Urea ↓, Pi ↓, H+ ↓ and serum proteins (=)	[5]
Kandouz et al.	138 patients in hemodiafiltration (HDF)	Vegan vs. non-vegan diet	Serum Indoxyl sulfate (IS) ↓ and p-cresyl sulfate (PCS) ↓	[8]
Rossi et al.	22 stage IV/V CKD patients	Symbiotic therapy	IS ↓, PCS ↓, renal parameters	[12]
Salmean at al.	13 CKD patients (≥50 mL/min/1.73 m^2^)	Cross-over low-fiber diet vs. high fiber diet	Renal parameters (↑ eGFR, BUN ↓, SCr ↓)	[13]
Khosroshahi et al.	50 ESRD patients on hemodialysis	Diet containing resistant stach vs. placebo	IS ↓, PCS ↓, Renal parameters (Urea ↓, Cr ↓, Uric acid ↓)	[18]
Goraya et al.	76 stage IV CKD patients	NaHCO3 vs. vegetable-based diet	Cystatin C =, UNAG↓, TGFβ =, aldoresterona ↑, tetrahidrocortisol/ tetrahidrocortisone ratio ↑, PTCO2 ↑	[35]
Goraya et al.	Macroalbuminuric CKD: Stage 1 (26 patients) and stage 2 (40 patients)	NaHCO_3_ vs. vegetable-based diet	Ualb ↑; UNAG =; TGFβ =; ET-1 ↓; Aldo ↑	[36]
Moe et al.	9 stage III/IV CKD patients	Vegetable-based diet vs. meat based diet for 7 days.	FGF-23(↑, PTH =, Ca =, Serum and urinary phosphate↓	[41]
Wu et al.	318 ESRD on hemodialysis	Vegetarians vs. non-vegetarians	nPCR ↓, Albumin =, antropometry (BMI↓, MACM↓) and hand grip =	[55]
Sirich et al.	56 ESRD on hemodialysis	Diet containing resistant stach vs. control starch	IS ↓, PCS ↓	[57]
Younes et al.	9 chronic renal failure patients	Fermentable carbohydrates suppementation (crossover)	Nutritional status and biochemistry (Urea ↓, Albumin =, pre-alb =)	[61]
Lu et al.	157 stage IV CKD patients	Dietary fiber correlation	ΔeGFR (slow), IL6 ↓, CRP ↓, IS ↓, SCh ↓	[99]
Saglinbene et al.	9757 ESRD patients on hemodialysis	Mediterranean and DASH diet scores	CV and total mortality (=)	[122]

Legend: ↑ Increase, ↓ Decreases, IS Indoxyl sulphate, PCS p-Cresol sulphate, MACM mid-arm muscular circumference; UNAG: Urinary N-acetyl-β-D-glucosaminidase; ET-1: Endotheline 1; Aldo: Aldosterone.
